# The XPF-ERCC1 Complex Is Essential for Genome Stability and Is Involved in the Mechanism of Gene Targeting in *Physcomitrella patens*

**DOI:** 10.3389/fpls.2019.00588

**Published:** 2019-05-09

**Authors:** Anouchka Guyon-Debast, Patricia Rossetti, Florence Charlot, Aline Epert, Jean-Marc Neuhaus, Didier G. Schaefer, Fabien Nogué

**Affiliations:** ^1^Institut Jean-Pierre Bourgin, INRA, AgroParisTech, CNRS, Université Paris-Saclay, Versailles, France; ^2^Laboratoire de Biologie Moléculaire et Cellulaire, Institut de Biologie, Université de Neuchâtel, Neuchâtel, Switzerland

**Keywords:** XPF-ERCC1, gene targeting, *Physcomitrella patens*, DNA repair, recombination

## Abstract

The XPF-ERCC1 complex, a highly conserved structure-specific endonuclease, functions in multiple DNA repair pathways that are pivotal for maintaining genome stability, including nucleotide excision repair, interstrand crosslink repair, and homologous recombination. XPF-ERCC1 incises double-stranded DNA at double-strand/single-strand junctions, making it an ideal enzyme for processing DNA structures that contain partially unwound strands. Here, we have examined the role of the XPF-ERCC1 complex in the model bryophyte *Physcomitrella patens* which exhibits uniquely high gene targeting frequencies. We undertook targeted knockout of the *Physcomitrella ERCC1* and *XPF* genes. Mutant analysis shows that the endonuclease complex is essential for resistance to UV-B and to the alkylating agent MMS, and contributes to the maintenance of genome integrity but is also involved in gene targeting in this model plant. Using different constructs we determine whether the function of the XPF-ERCC1 endonuclease complex in gene targeting was removal of 3′ non-homologous termini, similar to SSA, or processing of looped-out heteroduplex intermediates. Interestingly, our data suggest a role of the endonuclease in both pathways and have implications for the mechanism of targeted gene replacement in plants and its specificities compared to yeast and mammalian cells.

## Introduction

The XPF-ERCC1 complex is a highly conserved heterodimeric structure-specific endonuclease (SSE) composed of the XPF catalytic subunit and the ERCC1 DNA binding subunit that is involved in DNA repair and maintenance of chromosome stability (Dehé and Gaillard, 2017; Faridounnia et al., 2018). The protein sequences of the different ERCC1 homologs (ERCC1 in *Drosophila melanogaster*, Rad10 in *Saccharomyces cerevisiae* and Swi10 in *Schizosaccharomyces pombe*) and XPF homologs (MEI-9 in *D. melanogaster*, Rad1 in *S. cerevisiae* and Rad16 in *S. pombe*) are highly conserved as well as their capacity for heterodimerization, which insures stability and functionality of the complex. Consistent with the importance of heterodimerization of the two proteins for their function is that individual mutants in the *ERCC1* and *XPF* genes exhibit similar phenotypes (Gregg et al., 2011). The endonuclease activity of the XPF-ERCC1 complex is responsible for DNA cleavage near junctions between single-stranded and double-stranded DNA, where the single strand departs 5′ to 3′ from the junction.

XPF-ERCC1 is essential for nucleotide excision repair (NER) of DNA, a mechanism that removes DNA damage induced by ultraviolet (UV) radiation and by mutagenic chemicals, or chemotherapeutic drugs that form bulky DNA adducts. In mammalian cells, null mutants of the *ERCC1* or *XPF* genes are lethal and weaker mutations can result in xeroderma pigmentosum (XP), trichothiodystrophy (TTD), and Cockayne syndrome (CS), genetic disorders that are typical of mutations in genes required for NER (Gregg et al., 2011). However, there is evidence that the ERCC1 and XPF proteins have functions distinct from NER. Indeed, it was demonstrated that XPF-ERCC1 participates in the Fanconi Anemia Pathway of DNA interstrand crosslinks repair (Bhagwat et al., 2009) and recently the XPF-ERCC1 complex has been shown to be involved in a sub-pathway of long-patch base excision repair (BER) involving 5′ gap formation (Woodrick et al., 2017).

In addition to involvement in NER, BER and interstrand crosslink repair, there is evidence for a role of XPF-ERCC1 in double strand break (DSB) repair (Ahmad et al., 2008). Resolution of a DSB can be done by non-homologous end joining or by homology-directed repair (HDR) (Symington and Gautier, 2011). The XPF-ERCC1 complex and its *S. cerevisiae* homolog, the RAD1-RAD10 complex have been shown to participate to non-homologous repair of DSB and the RAD1 protein of *S. cerevisiae* and the mammalian ERCC1 protein play a major role in Alt-EJ (also known as MMEJ), a Ku-independent sub-pathway of NHEJ, that is error-prone, and that utilizes short stretches of homology to join two broken DNA ends (Ma et al., 2003; Ahmad et al., 2008). The RAD1-RAD10/XPF-ERCC1 complex participates also to intra or extra chromosomal HR between sequence repeats in *S. cerevisiae* (Klein, 1988; Schiestl and Prakash, 1988, 1990; Fishman-Lobell and Haber, 1992; Ivanov and Haber, 1995; Prado and Aguilera, 1995), in mammalian cells (Sargent et al., 1997, 2000; Al-Minawi et al., 2008) and in plants (Dubest et al., 2002, 2004). The function in HR of the RAD1-RAD10 endonuclease, is the removal of non-homologous 3′ termini of single-stranded overhangs of broken ends to facilitate single-strand annealing (SSA), an error-prone sub-pathway of HR (Bardwell et al., 1994; Pâques and Haber, 1997). Interestingly, the function of the RAD1-RAD10/XPF-ERCC1 complex has been shown to be also important for HR mediated gene targeting (GT) in *S. cerevisiae* and in mammalian cells (Schiestl and Prakash, 1988; Saffran et al., 1994; Adair et al., 2000; Niedernhofer et al., 2001; Langston and Symington, 2005; Rahn et al., 2011). There are two general methods for gene targeting, based on the two arrangements of donor DNA that can be used for gene targeting, called ends-in and ends-out (Hastings et al., 1993). They differ in whether the double-strand break is within the region of homology (ends-in) or at the ends (ends-out) leading to targeted gene replacement (TGR). Analysis of the capacity for GT using ends-in or ends-out substrates of recombination of mutants affected in the XPF-ERCC1 complex suggest that this complex could have different roles during gene targeting and that these roles could differ from one species to another.

In flowering plants, the study of the mechanisms of recombination is far behind that of mammals and yeast (Waterworth et al., 2011) and the low level of gene targeting efficiency, even if it can be increased to 1% by using a CRISPR-Cas9 based strategy (Wolter et al., 2018), makes the deciphering of this important mechanism difficult. However, in the plant kingdom the moss *Physcomitrella patens* is an exception to this rule and exhibits rates of gene targeting (GT) comparable to *S. cerevisiae* allowing advances in the understanding of DNA metabolism in plants (Schaefer and Zrÿd, 1997; Kamisugi et al., 2005, 2006; Odahara et al., 2007; Trouiller et al., 2007, 2006; Schaefer et al., 2010; Kamisugi et al., 2012; Wendeler et al., 2015; Collonnier et al., 2017; Odahara and Sekine, 2018; Wiedemann et al., 2018). It must be noticed that the mechanisms underlying targeted DNA integration are not necessarily conserved between *P. patens* and *S. cerevisiae* (Schaefer et al., 2010) and these differences can probably account for the differences that can be observed concerning the nature of the final products of ends-out constructs integration in the two species. First, ends-out construct integration producing single copy gene conversion (TGR), similar to what is observed in yeast, can be found in *Physcomitrella* but the majority (more than 80%) of the targeted integration events comprises head-to-tail concatemers of the transforming DNA fragment (Schaefer, 2002; Kamisugi et al., 2006), that could result from episomally replicating DNA (Murén et al., 2009). Second, a significant proportion (around 50%) of gene targeting events in *Physcomitrella* result in insertion of the ends-out construct adjacent to the target locus (targeted gene insertion-TGI). Typically, TGI comprises an HR event at one end of the integrant, accompanied by an apparent NHEJ event at the other (Schaefer, 2002; Kamisugi et al., 2005, 2006; Collonnier et al., 2017). This profile of integration is rarely observed in *S. cerevisiae* but frequent for GT events in flowering plants and animal cells (Adair et al., 1998; Hanin et al., 2001).

To investigate the potential role of the XPF-ERCC1 complex in DSB repair and gene targeting mechanisms in *Physcomitrella* we produced mutants for the *PpERCC1* and *PpXPF* homologous genes. Our data show that loss of ERCC1 and/or XPF functions generates a strong mutator phenotype and hypersensitivity to UV-B and methyl methanesulfonate (MMS) induced DNA lesions, suggesting an active role of this complex in the moss NER and BER repair pathways. Using different constructs we further revealed that the moss XPF-ERCC1 complex is required for HR between both ends-out or ends-in constructs and genomic loci.

## Materials and Methods

### Plant Material and Growth Conditions

*Physcomitrella patens* (Hedw.) B.S.G. “Gransden” was used in this study. Individual strains were vegetatively propagated as lawns of protonemal filaments on rich agar PpNH_4_ medium (PpNO_3_ medium supplemented with 2.7 mM NH_4_-tartrate) overlaid with cellophane, or cultured as “spot inocula” on minimal medium (PpNO_3_) for phenotypic analyses and sporogenesis as previously described (Trouiller et al., 2006). Protoplast isolation, PEG mediated transformation and selection of transformed plants were performed according to (Schaefer et al., 2010).

### Gene Identification and Isolation

Genomic DNA and total RNA were isolated from *Physcomitrella* as previously described (Trouiller et al., 2006). *Physcomitrella* genomic sequences encoding the *ERCC1* and *XPF* genes were identified by BLAST search^1^. The available gene models were used for the design of PCR primers to amplify cognate genomic sequences, which were cloned in the TOPO^®^-TA (life technologies, United States) or pBluescript (Stratagene, United States) plasmids. PCR primers used are listed in Supplementary Table S1. In order to obtain a correct gene model for each sequence, full-length cDNA were amplified from *Physcomitrella* polyribosome-derived RNA by RT-PCR (Kamisugi et al., 2012) and sequenced. Predicted polypeptide sequences were aligned with the orthologous genes from other eukaryotes using CLUSTALW.

### Generation of Deletion Mutants

The KO vector pERCC1 delta contains a 654 bp 5′-targeting fragment (chrom6: 19338880–19339533) and an 865 bp 3′-targeting fragment (chrom6: 19341999–19342863) flanking a LoxP-HygroR-LoxP marker in vector pBHRF (Schaefer et al., 2010). The KO vector pXPF delta contains a 974 bp 5′-targeting fragment (chrom18: 8489087–8490060) and a 1096 bp 3′-targeting fragment (chrom18: 8493588–8494683) flanking a LoxP-NeoR-LoxP marker in vector pBNRF (Schaefer et al., 2010). Moss protoplasts were transformed with pERCC1 delta digested with *Avr*II and *Pac*I, or with pXPF delta digested with *Xba*I and *Pac*I. Transformed plants carrying targeted gene replacement were identified by PCR genotyping and subsequent deletion of the selection marker was obtained by transient Cre recombinase expression (Trouiller et al., 2006). The double *ercc1/xpf* mutant was generated by retransforming a *PpErcc1*Δ deletion line with pXPF delta and selecting for targeted gene replacement at the *PpXPF* locus among neo^*R*^ plants.

### Analysis of Gene Expression in Mutants

Transcript abundance in selected knockout lines was determined by RT-PCR. Total RNA was extracted using TRIZOL-reagent (Invitrogen) from 100 mg of protonemal tissue. Contaminating DNA was removed by DNaseI treatment with RNase Free DNase set (Qiagen) using spin columns of the Rneasy plant mini kit (Qiagen). RT-PCR was performed with RevertAid H Minus M-MuLV Reverse Transcriptase (Fermentas) on 500 ng RNA according to the supplier’s instructions. Quality control of DNA or RNA was performed using primers PpAPT#14 + PpAPT#19 (Supplementary Table S1). Detection of *ERCC1* and *XPF* mRNA in mutant lines was performed by RT-PCR using primers indicated in Supplementary Table S1.

### UV-B and MMS Sensitivity Assays

For UV-B sensitivity protoplasts of wild-type, *ercc1*Δ, *xpf*Δ, or *xpfK*Δ/*ercc1*Δ strains were spread (ca. 25000/plate) and regenerated on protoplast agar medium (PpNH_4_ + 0.5 g/L glucose + 6.6% mannitol). Plates were immediately exposed to UV-B light (60 J/m^2^/s) from a 312 nm TFX lamp. We calculated the flux with a UV-Elektronik GmbH dosimeter. The. Plates were immediately transferred to darkness for 24 h after treatment then to standard growth conditions for protoplast regeneration. Survival was determined as described previously (Kamisugi et al., 2012). For Methyl methanesulfonate (MMS) (Sigma-Aldrich) sensitivity, protoplasts were spread on protoplast agar medium freshly supplemented with different concentration of MMS and further regenerated under standard growth conditions. Cultures were transferred to PpNH_4_ medium without MMS after 6 days and survival was determined 2 weeks later by microscopic observation.

### Evaluation of Spontaneous Mutation Frequency

To assess the mutator phenotype of *ercc1*Δ and *xpf*Δ mutants, we measured the level of spontaneous loss of function of the Adenine Phosphoribosyl Transferase gene (*PpAPT*) which confers resistance to 2-fluoroadenine (2-FA), as previously described (Trouiller et al., 2006). Several million of plants were regenerated from protoplasts of wild-type, *ercc1*Δ and *xpf*Δ mutants and then transferred to medium supplemented with 10 mM 2-FA (Fluorochem). After 2 weeks, the number of resistant plants was counted. Results were analyzed using the Fisher’s exact test.

### Gene Targeting Assays

The different APT based gene targeting constructs used in this study are described in Figure 3, 4. GT efficiencies were assessed after transformation with vector PpAPT-KO2 (Schaefer et al., 2010) and selection for Hygro^*R*^ plants followed by selection for 2-FA^*R*^ plants among them, as performed in Charlot et al. (2014), GT frequencies were assessed after transformation with vector PpAPT-KO2 or PpAPT-KO9 and direct selection of 2-FA^*R*^ plants, a selection that only identifies targeted integration events in *PpAPT*. To evaluate the importance of the form of the transforming DNA, transformation was performed with PpAPT-KO9 digested with *Eco*RI + *Hin*dIII (ends-out) or with *Xba*I (ends-in) and direct selection for 2-FA^*R*^ plants. To study the impact of heterologous sequences at the ends of the transforming DNA, transformation was performed with PpAPT-KO9 digested with *ApaL*I or with PpAPT-KO2 linearized within the hygromycin resistance cassette with *AsiS*I (long ends-in): in both cases transformation was followed by direct selection for 2-FA^*R*^ clones. Experiments were repeated three to eight times and statistically analyzed using the Fisher exact test.

### Analysis of Transformed Plants

In order to analyze large numbers of transformed plants for the nature of gene targeting events, PCR-based genotyping assays were used. Primers PpAPT#2 and PpAPT#20 located outside the genomic fragments present in the cassette (Supplementary Table S1 and Figure 3) were used to detect monocopy insertions. For detection of targeted gene replacement (TGR) and targeted gene insertion (TGI) integrations the 5′ and 3′ junctions of the integrations were characterized using primers PpAPT#2 + ProRev and PpAPT#20 + TerFwd (Supplementary Table S1 and Figure 3) respectively.

### Cytology

Binocular observations were made with a Nikon SMZ1000.

## Results

### Identification of PpXPF and PpERCC1 and Generation of Deletion Mutants

**FIGURE 1:**
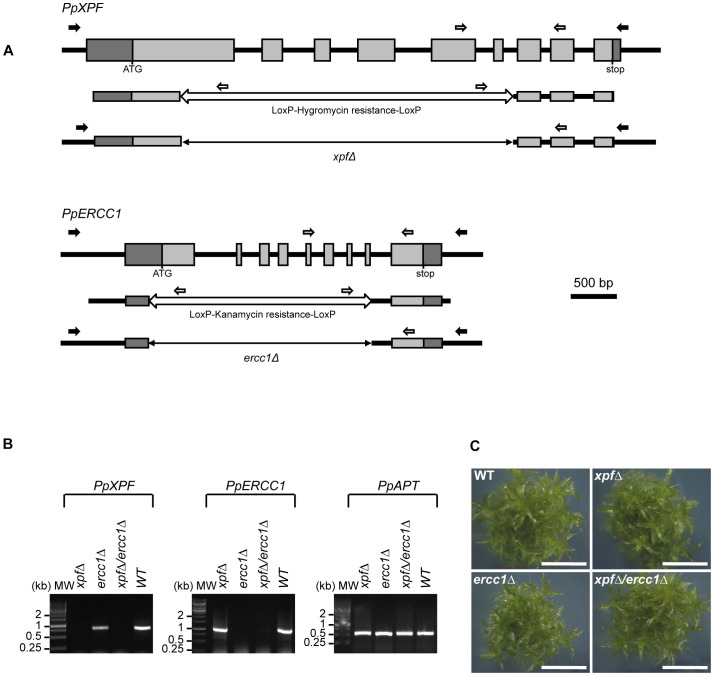
Structure and targeted disruption of *Physcomitrella XPF* and *ERCC1* genes. **(A)** Structure of the WT, *PpXPF*, and *PpERCC1* loci (top line), of the KO vector (middle) and of the deleted locus in the *xpf*Δ and *ercc1*Δ mutants (bottom). Exons are represented by gray boxes, with 5′- and 3′-UTR sequences in darker gray. The region deleted by cre-lox excision of a selection cassette is shown as a double arrow line. Arrows indicate the position of the primers used to genotype the plants by PCR (black and white) and RT-PCR (gray). **(B)** RT-PCR analysis of *XPF* and *ERCC1* transcripts in wild-type and mutants plants. RNA was isolated from protonemal tissue of wild-type and mutants lines for cDNA synthesis and PCR amplification using gene-specific primers. The *PpAPT* transcript has been used as control. Primers are listed in Supplementary Table S1. **(C)** Morphology of plants of wild type of *xpf*Δ, *ercc1*Δ single mutants and *xpf*Δ/*ercc1*Δ double mutant. The picture was taken after 3 weeks of growth. scale bar = 4 mm.

The XPF-ERCC1 heterodimeric complex is a highly conserved structure endonuclease (Schwartz and Heyer, 2011). Sequence homology searches of the *P. patens* genome with the Arabidopsis homologs identified a single putative homolog for XPF (*PpXPF*, Pp3c18_11670) and ERCC1 (*PpERCC1*, Pp3c6_29610). PpXPF and PpERCC1 full length cDNAs were isolated and sequenced: this analysis confirmed the predicted structures (9 exons, Figure 1A) and protein sequences found in the database (Phytozome 12.0). A phylogenetic analysis, including plants, algae, animals and fungi established that *PpXPF* and *PpERCC1* effectively belong to the XPF and ERCC1 plant clades among eukaryotic SSE of the XPF/MUS81 family (Supplementary Figure S1). The moss PpXPF protein is composed of 1047 amino acids and shares 50.3, 35.5, and 30.4% sequence identity with AtXPF, HsXPF, and SpRad16, respectively (Supplementary Figure S2). The homology between PpXPF and the XPF/RAD1 homologs is distributed throughout their length, but it is especially strong at the C-terminal end, in the region involved in the formation of the heterodimeric protein complex. Noticeably, the nuclease domain (restriction endonuclease type-II like PR011335) containing the specific motif ERKXXXD required for nuclease activity (Ciccia et al., 2008) and the RuvA_2-like domain (IPR010994) containing the duplicated Helix-hairpin-Helix (HhH) motif (PFAM:HHH_5) functioning as a scaffold for complex formation with ERCC1 (Tripsianes et al., 2005) can be identified in PpXPF (Supplementary Figure S2). The moss PpERCC1 protein is composed of 427 amino acids and shares 44.6, 27.6, and 23.3% sequence identity with AtERCC1, HsERCC1, and SpSwi10, respectively (Supplementary Figure S3). Sequence conservation is especially high within the central RAD10 domain (IPR004579) and the C-terminal RuvA 2-like domain, which are involved in DNA binding and complex formation in ERCC1 homologs (Manandhar et al., 2015). These features are consistent with our assumption that PpXPF and PpERCC1 encode the proteins forming the moss heterodimeric XPF/ERCC1 complex involved in several aspects of DNA repair.

To investigate PpXPF and PpERCC1 functions we generated *xpf* and *ercc1* deletion mutants, named *xpf*Δ and *ercc1*Δ respectively, using targeted gene disruption followed by Cre/lox mediated elimination of the resistance cassette (Figure 1A and section “Materials and Methods”). Deletion of exons 1–8 in *PpERCC1* and of exons 1–6 in *PpXPF* was confirmed by PCR genotyping and sequence analysis (data not shown). A double knock-out mutant named *xpf*Δ/*ercc1*Δ was produced by re-transformation of an *ercc1*Δ mutant with the pXPF delta vector. RT-PCR analysis established that the full-length transcripts were no longer produced in these mutants (Figure 1B). For all further experiments, we used two independent *xpf*Δ, *ercc1*Δ, or *xpf*Δ/*ercc1*Δ strains and both alleles of the same mutants show similar phenotypes.

### The *xpf*Δ, *ercc1*Δ, and *xpf*Δ/*ercc1*Δ Mutants Show No Developmental Defects

In Bryophytes, the life cycle is dominated by the haploid gametophyte. Haploid spores of *P. patens* germinate to form a juvenile filamentous network of tip growing cells, the protonema. One week after germination, initials of leafy shoots called buds differentiate from protonemal branch initials and further develop by meristematic growth into the leafy gametophores. After 1 month, each individual plant is composed of several dozens of gametophores. Short day length and low temperature induces the differentiation of the reproductive organs at the shoot apex. Finally fertilization of the egg cell by flagellated antherozoids give rise to the epiphytic diploid sporophyte in which meiosis takes place to produce new haploid spores, reviewed in Bonhomme et al. (2013) and Kofuji and Hasebe (2014).

The phenotype of the *xpf*Δ, *ercc1*Δ, and *xpf*Δ/*ercc1*Δ mutants was assessed throughout the entire life cycle. Protonemal growth, bud differentiation and leafy shoots development were similar in the three mutants compared to the wild-type and the *xpf*Δ, *ercc1*Δ, and *xpf*Δ/*ercc1*Δ strains are fertile (Figure 1C and Supplementary Figure S4). These findings show that the XPF/ERCC1 complex plays no direct role in either vegetative or reproductive moss development.

### The *xpf*Δ, *ercc1*Δ, and *xpf*Δ/*ercc1*Δ Mutants Are Impaired in Repairing Exogenous and Endogenous DNA Damage

**FIGURE 2:**
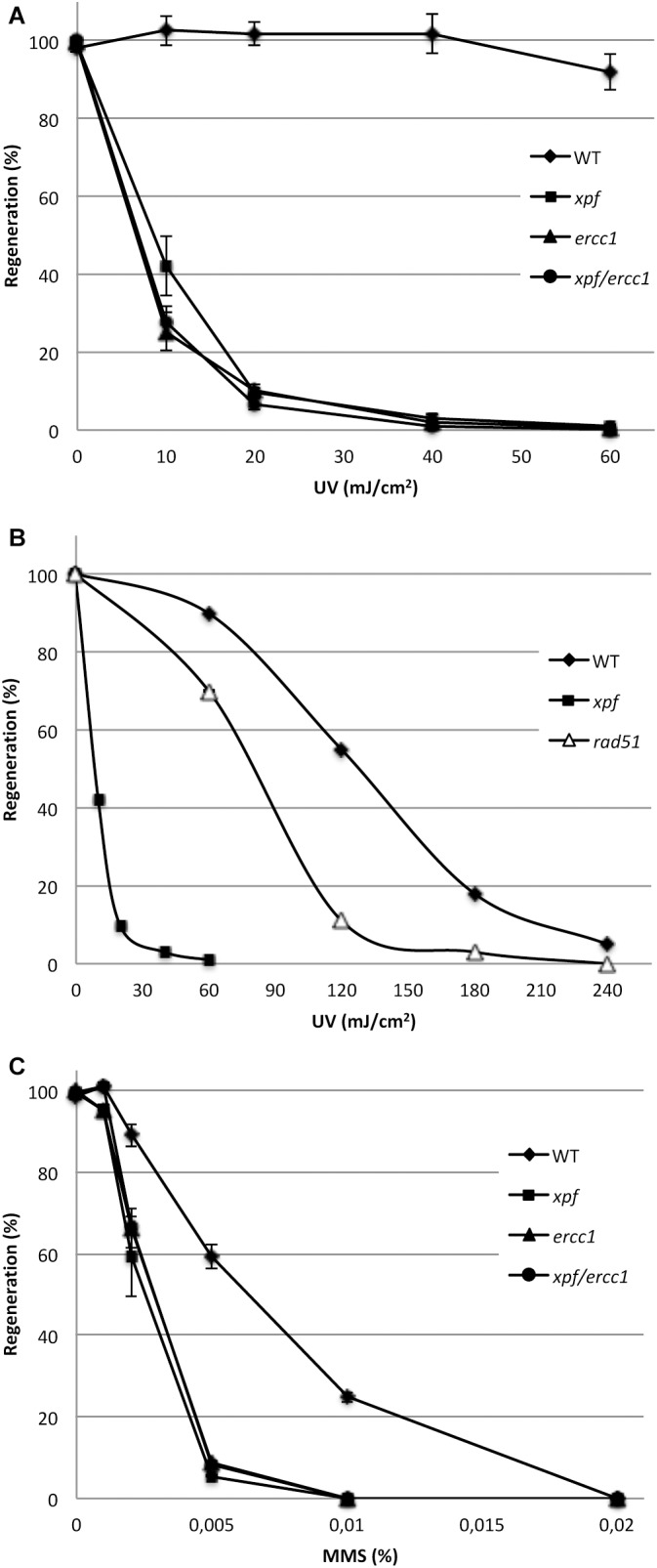
Sensitivity of the wild-type (WT) and of the *xpf*, *ercc1*, and *xpf/ercc1* mutants toward genotoxic agents. **(A)** Survival curves of the WT, *xpf* and *ercc1* (*n* = 4), and *xpf/ercc1* strains (*n* = 2) in response to low doses of UV-B light (scale bar = standard deviation). **(B)** The survival curves of WT, *xpf* and *rad51* strains in response to higher UV-B doses are shown (references: this study and Charlot et al., 2014). **(C)** Survival rates of protoplasts after exposure to MMS: survival is expressed as the percentage of regenerated protoplasts relative to untreated samples. The experiment was performed two times except for *xpf*/*ercc1* (scale bar = standard deviation).

The XPF/ERCC1 complex is involved in several DNA repair pathways in eukaryotes: it is a major factor of NER and also contributes to ICL, SSA, and HR. We therefore evaluated the sensitivity of the mutants to DNA damage induced by UV-B and methyl methanesulfonate (MMS). To assess the sensitivity of the *xpf*Δ, *ercc1*Δ, and *xpf*Δ/*ercc1*Δ strains to UV-B light, which generates DNA damage essentially repaired by the NER pathway, we monitored the ability of UV-B-treated protoplasts to regenerate into plants. Our data show that both *xpf*Δ and *ercc1*Δ mutants display an extremely high UV-B sensitivity which is not further increased in the double *xpf*Δ/*ercc1*Δ strain (Figure 2 and Supplementary Figure S5). With a lethal dose 50 around 8 mJ/cm^2^, these mutants are ca. 10-fold more sensitive than the *rad51-1-2 double mutant* strain (Schaefer et al., 2010; Charlot et al., 2014) and 20-fold more sensitive than the WT (Figure 2). Such a strong sensitivity to UV-B provides evidence for a key involvement of the moss XPF/ERCC1 complex in the repair of UV-induced DNA damage, most likely by the NER pathway. We further evaluate the implication of the XPF/ERCC1 complex in the repair of DNA damage induced by the alkylating agent MMS, which is believed to stall replication forks. In this assay, fresh protoplasts are regenerated for 6 days on MMS containing medium, and their ability to regenerate into plants is evaluated after a further 14-days of growth on standard medium. Our analyses show that the *xpf*Δ, *ercc1*Δ, and *xpf*Δ/*ercc1*Δ strains display a similar increased sensitivity to MMS compared to the WT (Figure 2 and Supplementary Figure S5). This finding indicates that the moss XPF/ERCC1 complex contributes to the repair of DNA damages induced by an MMS treatment. Further experiments are needed to characterize the exact nature of these damages.

Finally to evaluate the level of contribution of the XPF/ERCC1 complex to the repair of endogenous DNA damage, we measured the level of spontaneous loss of function of the *PpAPT* reporter gene (Trouiller et al., 2006) and assess the mutator phenotype of these mutants. More than 2 million protoplasts of WT, *xpf*Δ and *ercc1*Δ strains were regenerated and selected for their resistance to 2-fluoroadenine. No 2-FA resistant plants were recovered in the WT, and a total of 5 and 4 2-FA resistant plants were identified in the *xpf*Δ and *ercc1*Δ mutant, respectively (Table 1). We previously showed that the *APT* mutation rate in WT was around 10^−8^ (Charlot et al., 2014), this analysis shows that the *APT* mutation rate is increased approximately 100-fold in the *xpf*Δ (2.5 × 10^−6^) and *ercc1*Δ (1.8 × 10^−6^) strains. These data support a direct involvement of the moss XPF-ERCC1 complex in repairing naturally occurring DNA damage to prevent the accumulation of mutations in the genome. Taken together the above data demonstrate that the moss XPF-ERCC1 complex plays an important role in the repair of both endogenous and exogenous DNA damage.

**Table 1:** PpXPF and PpERCC1 are required to repair endogenous DNA damage.

Genotype	Regenerants (×10^3^)^a^	2-FA resistant	Rate in 10^6^
WT	107200	2	0.02
*xpf*Δ	1963	5 (*p* = 3.8 × 10^−8^)^b^	2.54
*ercc1*Δ	2216	4 (*p* = 2.4 × 10^−6^)^b^	1.89

*^a^Regenerants number was evaluated 5 days after protoplasts isolation, before transfer on 2FA. ^b^Differences between WT and mutants were compared using Fisher’s exact test.*

### Gene Targeting Using an Ends-Out Construct Is Reduced in the *xpf*Δ and *ercc1*Δ Mutants

**FIGURE 3:**
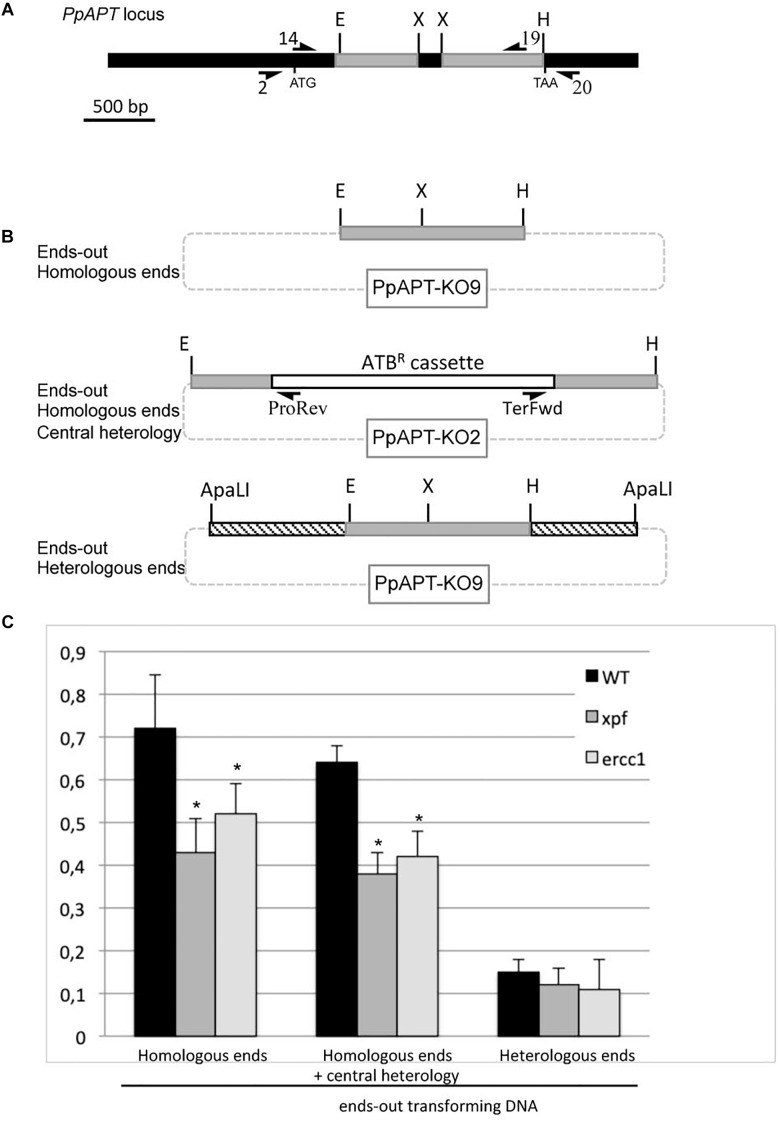
Gene targeting frequency in WT and *xpf* and *ercc1* mutants using ends-out type vectors. **(A)** PpAPT WT locus. Regions targeted using ends-out or ends-in vectors are in gray. Primers used in this study are referenced in Supplementary Table S1. Different forms of the transforming DNA used. **(B)** Digestion of PpAPT-KO9 with *Eco*RI + *Hin*dIII give rise to ends-out transforming DNA, with double strand breaks at the edges of *APT* sequences. Digestion of PpAPT-KO2 with *Eco*RI + *Hin*dIII give rise to ends-out transforming DNA, with double strand breaks at the edges of *APT* sequences and a central heterologous region. Digestion of PpAPT-KO9 with *ApaL*I generates ends-out transforming DNA with heterologous sequence at the DSB (dashed bars: heterologous stretches, thin lines: plasmid sequences, dotted lines: plasmid sequences absent from the transforming DNA). **(C)** Gene targeting frequency of the *APT* gene in the wild type, and *xpf* and *ercc1* mutants using different ends-out types transforming DNA. Asterisks indicate significant differences with the WT (Fisher exact test *p*-value ≤0.01).

In yeast and mouse ES cells, the RAD1-RAD10 (or XPF-ERCC1) complex is involved in gene targeting using ends-out constructs (Niedernhofer et al., 2001; Langston and Symington, 2004, 2005). In order to investigate the involvement of the moss XPF-ERCC1 complex in gene targeting, we determined gene targeting rates in wild-type, *xpf*Δ and *ercc1*Δ mutants cells after transformation with an ends-out targeting substrate with homologous ends designed to inactivate the *PpAPT* gene and containing an hygromycin resistance cassette (PpAPT-KO2) (Figure 3). The gene targeting efficiency (GTE), determined as the frequency of 2-FA resistant plants amongst transformed plants (Hyg^*R*^) (Table 2), reaches 69.5% in the wild-type and is reduced by 1.7 and 1.5-folds in the *xpf*Δ, and *ercc1*Δ mutants respectively (Table 2, Fisher’s test *p* ≤ 0.003). These experiments demonstrate that PpXPF and PpERCC1 contribute to but are not essential for gene targeting.

**Table 2:** Comparison of transformation and gene targeting efficiencies using an ends-out type targeting construct containing an heterologous selectable marker.

Genotypes	Regenerants^a^	Hyg^*R*^ plants	2FA^*R*^ plants^b^	Gene targeting frequency (%)^c^	Gene targeting efficiency (%)^d^
Wild type	94219	868	603	0.64 ± 0.08^e^	69.5 ± 9.57^e^
*xpf*	124210	1091	472^∗^	0.38 ± 0.1	43.3 ± 12.42
*ercc1*	22381	205	94^∗^	0.42 ± 0.12	45.8 ± 16.32

*^a^Protoplasts were transformed with vector PpAPT-KO2 digested with BsaAI + BsrGI (ends-out) and regenerants were selected on hygromycin. ^b^2-FA^*R*^ clones are the stable Hyg^*R*^ clones that experienced a gene targeting event and thus survived after subculture on 2-FA medium. Differences between wild type and the mutants were compared using Fisher’s exact test. ^∗^Correspond to p-value <0.01. ^c^GTF (in %) express the frequency of 2-FA resistant among the population of regenerants. ^d^GTE (in %) express the frequency of 2-FA resistant among the population of stably transformed plants (Hyg^*R*^). ^e^Average and standard deviation were determined from at least two independent experiments.*

### Gene Targeting Decrease in the *xpf*Δ and *ercc1*Δ Mutants Is Not Due to the Heterologous Selectable Marker

The linearized ends-out targeting constructs PpAPT-KO2 used in the previous experiment does not contain non-homologous tails at its 3′ ends. For this reason the decrease of GT in the *xpf*Δ or *ercc1*Δ mutants cannot be attributed to the role of the PpXPF-PpERCC1 complex in removing 3′ overhangs of non-homologous sequences as described for SSA or for targeted integration of ends-in targeting constructs in CHO cells. Nevertheless, in yeast and mouse the endonuclease complex has been shown to be also involved in the resolution of the large loop of mismatches that forms between the targeted gene and the central region of heterology corresponding to the selectable marker (Niedernhofer et al., 2001; Langston and Symington, 2004, 2005). In order to determine if the moss XPF-ERCC1 complex has a role in handling the heterology generated by the presence of the selectable marker (hygromycin resistance cassette in this case), GT frequencies (GTF) of an ends-out targeting substrate with homologous ends but lacking a selection marker (Figure 3, PpAPT-KO9 digested by *Eco*RI/*Hin*dIII), were measured. This substrate confers 2-FA resistance upon targeted integration at the *PpAPT* locus, generating a 159 bp deletion. The gene targeting frequency was determined as the frequency of 2-FA resistant plants amongst regenerated protoplasts (Table 2 and Supplementary Table S2). GTF observed in the wild type using this ends-out targeting construct is slightly but significantly higher (Fisher exact test *p*-value = 0.01) compared to GTF observed using the ends-out targeting construct containing the selection marker that creates a central region of heterology (Figure 3). GTFs observed using this ends-out targeting construct in the *xpf*Δ and *ercc1*Δ mutants were reduced by 1.7 and 1.4-fold respectively compared to wild type (Figure 3). This reduction is very similar to the one observed using the ends-out targeting construct containing the selectable marker. These results show that the ERCC1 and XPF proteins are necessary for gene targeting of an ends-out type targeting construct even in the absence of a large loop of mismatches in its central region.

### Gene Targeting Using an Ends-In Construct Is Affected in the *xpf*Δ and *ercc1*Δ Mutants

**FIGURE 4:**
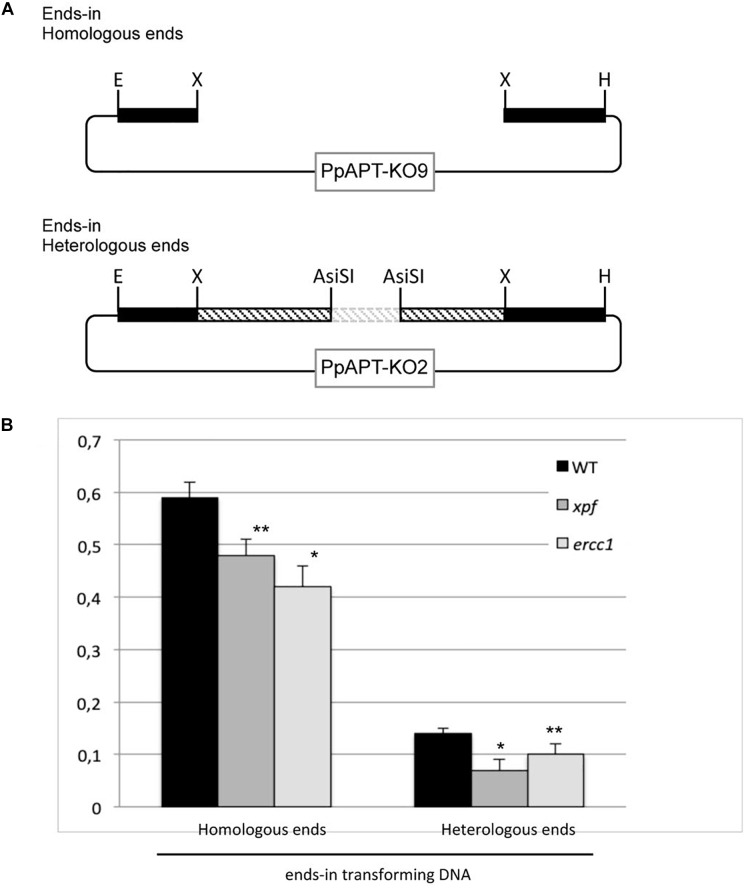
Gene targeting frequency in WT and *xpf* and *ercc1* mutants using ends-in type vectors. **(A)** Different forms of the transforming DNA used. Digestion of PpAPT-KO9 with *Xba*I give rise to ends-in transforming DNA, with double strand breaks at the edges of *APT* sequences. Digestion of PpAPT-KO2 with *AsiS*I generates ends-in transforming DNA with heterologous sequence at the double strand breaks (dashed bars: heterologous stretches, thin lines: plasmid sequences, dotted lines: plasmid sequences absent from the transforming DNA). **(B)** Gene targeting frequency of the *APT* gene in the wild type, and *xpf* and *ercc1* mutants using different ends-in types transforming DNA. Asterisks indicate significant differences with the WT (^∗^Fisher exact test *p*-value ≤0.01; ^∗∗^Fisher exact test *p*-value ≤0.05).

In *S. cerevisiae* and in hamster cells (CHO), the RAD1-RAD10/XPF-ERCC1 is involved in removing long non-homologous tails from the 3′ ends of invading strands from ends-in gene targeting constructs (Schiestl and Prakash, 1988, 1990; Adair et al., 2000; Sargent et al., 2000). In Arabidopsis, XPF (RAD1) and ERCC1 (RAD10) have been shown to play a role in intermolecular recombination between plasmids by removing non-homologous 3′ ends from recombination intermediates (Dubest et al., 2002, 2004). In order to test the role of the moss XPF-ERCC1 complex in gene targeting using an ends-in targeting substrate with homologous ends GT frequencies (GTF) of an ends-in targeting construct (Figure 4, PpAPT-KO9 digested by *Xba*I), were measured in the wild type and *xpf*Δ or *ercc1*Δ mutants. GTF observed in the wild type using this ends-in targeting construct is slightly (1.2-fold) but significantly lower (Supplementary Table S2, Fisher exact test *p*-value = 0.014) compared to GTF observed using the ends-out targeting construct. GTF, reaches 0.59% in the wild-type and is reduced by 1.2 and 1.4-folds in the *xpf*Δ, and *ercc1*Δ mutants respectively (Figure 4 and Supplementary Table S2, Fisher’s test *p*-value ≤0.05). Use of an ends-in targeting substrate with heterologous ends strongly decrease the GTF in the wild type and the two mutants (Figure 4 and Supplementary Table S2). These results show that the ERCC1 and XPF proteins are also involved in gene targeting of an ends-in type targeting construct and that presence of heterologous sequences at the 5′ and 3′ extremities of this type of construct is very detrimental for the efficiency of gene targeting.

### The Nature of Targeted Integration Is Modified in the *xpf*Δ and *ercc1*Δ Mutants

**FIGURE 5:**
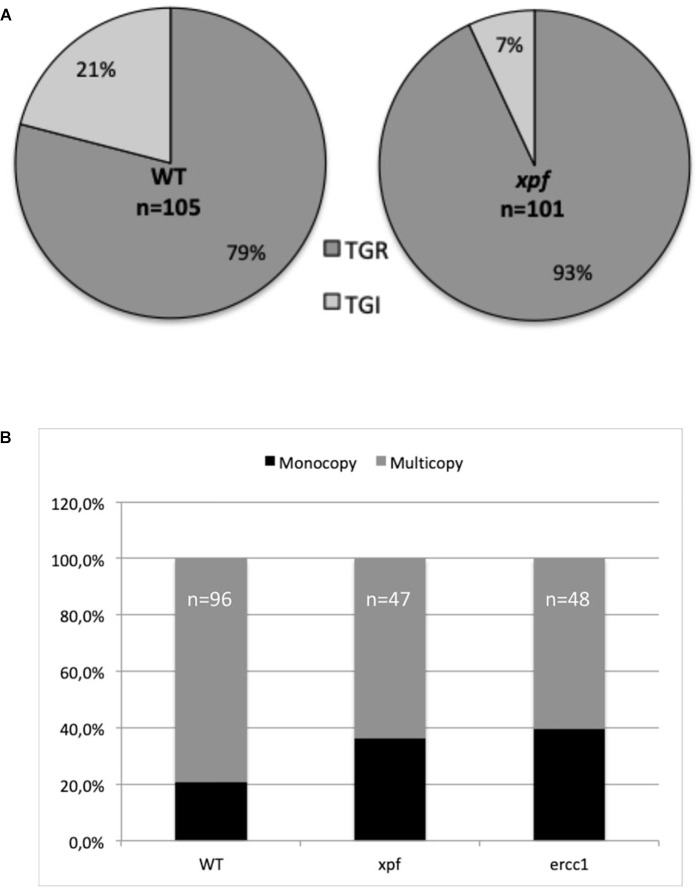
Type of integrations at the *APT* locus using an ends-out construct for the wild-type (WT) and mutants. **(A)** Rate of targeted gene replacement (TGR) vs. targeted gene insertion (TGI) integrations was estimated using primers specific to the PpAPT-KO2 cassette and primers located on the *PpAPT* gene but outside the genomic fragments present on the donor DNA cassette in WT and *xpf* mutant (see section “Materials and Methods,” Figure 3 and Supplementary Figure S6). **(B)** Rate of monocopy vs. multicopy insertions at the targeted locus was estimated in WT and *xpf* and *ercc1* mutants, using primers located outside the sequences homologous to the gene fragments present in the PpAPT-KO9 donor DNA template (see section “Materials and Methods,” Figure 3 and Supplementary Figure S6).

If, as in yeast, *P. patens* stable transformants can result from a double recombination at both ends of the ends-out type targeting fragment leading to TGR, another type of integration, named targeted gene insertions (TGI, Supplementary Figure S6), can be found in *P. patens*, like in ES cells (Kamisugi et al., 2006). In order to test the role of the PpXPF-PpERCC1 complex in the formation of TGI, we measured, using a PCR based approach, the ratio of targeted gene replacement (TGR) versus targeted gene insertion (TGI) in 2FA^*R*^ plants obtained after transformation with the ends-out targeting substrate (PpAPT-KO2 digested *Eco*RI/*Hin*dIII, Table 2 and Figure 3) in the wild-type and *xpf* backgrounds. For the wild-type, 79% of targeted transformants were identified as TGR and 21% as TGI, while in the *xpf*Δ mutant, a statistically higher number of the transformants (Fisher’s exact test *P* = 0.005) were identified as TGR (93%) and 7% as TGI (Figure 5A). These findings show that XPF and probably the XPF-ERCC1 complex is an important factor in the mechanism leading to TGI in *P. patens*.

Another marked difference between *S. cerevisiae* and *P. patens* concerning the type of integration is the fact that insertion of concatenated copies of the donor cassettes is frequent in GT experiments in *P. patens* (Supplementary Figure S6; Kamisugi et al., 2006). These concatenates result probably from episomally replicating DNA (Murén et al., 2009) a characteristic that have been used recently for complementation of an auxotrophic marker in *P. patens* (Ulfstedt et al., 2017). In order to clarify the role of the PpXPF-PpERCC1 complex in the formation of targeted gene replacement with head-to-tail multicopy, we measured, using a PCR based approach, the number of monocopy TGR (Supplementary Figure S6) in 2FA^*R*^ plants obtained after transformation with the ends-out targeting substrate (PpAPT-KO9 digested *Eco*RI/*Hin*dIII, Figure 3) in the wild-type and mutants backgrounds. The percentage of monocopy integration in the WT and in the *xpf*Δ and *ercc1*Δ mutants was 21, 36, and 40% respectively (Figure 5B). Therefore the proportion of monocopy TGR is significantly more important in the *xpf*Δ and *ercc1*Δ mutants compared to the wild-type (chi-squared test *P* < 0.05).

We can conclude from these results that the nature of the targeted integrations is altered in these mutants. The PpERCC1 and PpXPF proteins are involved in the mechanism that results in the integration of the ends-out targeting construct via TGI and in the integration of concatemers. Nevertheless, TGI type integrations and concatemers integrations can be detected in the mutants contexts, implying that other nucleases can partially complement the absence of XPF and ERCC1 for these mechanisms in *P. patens*.

## Discussion

We report here the identification and characterization of the *P. patens* mutant for the *XPF* and *ERCC1* genes. The *xpf* and *ercc1 P. patens* mutants are viable and show no phenotypic defect under normal conditions, in agreement with what was observed for the Arabidopsis *xpf* and *ercc1* mutants (Hefner et al., 2003; Preuss and Britt, 2003; Dubest et al., 2004), but in contrast with the situation observed in mammalian cells, where null mutants of the ERCC1 or XPF genes are lethal (McWhir et al., 1993; Núñez et al., 2000; Hsia et al., 2003; Tian et al., 2004). Like their Arabidopsis counterparts the *xpf* and *ercc1 P. patens* mutants are fully fertile suggesting that in plants, like in *S. cerevisiae*, this complex has only a minor role, if one, in meiosis which is in contrast with what is observed in Drosophila or *C. elegans* where the homologs of XPF1 have been shown to be involved in meiotic crossover formation (Sekelsky et al., 1995; Radford et al., 2005; Agostinho et al., 2013; O’Neil et al., 2013; Saito et al., 2013).

The *P. patens xpf* and *ercc1* mutants present a strong increase in sensitivity to UV-B compared with the wild type. These results are consistent with previous studies on this complex in yeast (Prakash et al., 1993), animals (Gregg et al., 2011), and in *Arabidopsis thaliana* (Fidantsef et al., 2000; Liu et al., 2000; Hefner et al., 2003; Dubest et al., 2004; Biever et al., 2014) and confirm the requirement of the XPF-ERCC1 complex in the NER pathway in plants. Interestingly, the frequency of spontaneous mutations (mutator rate) in the *P. patens xpf* and *ercc1* mutants is very high compared to wild type (100-fold increase) and is even higher than the one found in the *rad51* mutant background, depleted for homologous recombination (Schaefer et al., 2010). This is reminiscent of what is observed in the *S. cerevisiae rad1* mutant where an increases in the frequencies of single-base-pair substitution, single-base-pair deletion and insertion of the yeast retrotransposon Ty have been described (Kunz et al., 1990). It must be noticed that the fold increase in spontaneous mutations in the *xpf*/*rad1* mutants backgrounds is significantly higher in *P. patens* compared to *S. cerevisiae* (Kunz et al., 1990; Doetsch et al., 2001). These data demonstrate the essential role of the XPF/ERCC1 complex in genome stability in *P. patens* and could potentially reflect a more prominent role of the NER pathway in genome stability in *P. patens* compared to *S. cerevisiae*. In addition, we have shown here that XPF-ERCC1 is also important for the response to MMS and the recent observation in *P. patens* that the *rad51* mutant is more sensitive to MMS compared to WT (Goffová et al., 2019) reinforces the hypothesis that the HR machinery, and the XPF-ERCC1 complex would contribute to the repair of damages that would result from an MMS treatment, the exact nature of these damages being still unclear (Wyatt and Pittman, 2006). The role of the *P. patens* XPF/ERCC1 complex in these repair pathways could also be the cause of the important genetic instability observed in the corresponding mutants background.

There is good evidence for a role of XPF-ERCC1 in repair of double strand break through homologous recombination (Ahmad et al., 2008). We show here that the *P. patens* XPF-ERCC1 complex is involved in gene targeting using an ends-in construct and is also, and potentially even more important, for gene targeting using an ends-out construct. Concerning the ends-in construct the role of the *P. patens* XPF-ERCC1 complex could be, as in *S. cerevisiae* and in hamster cells (CHO), to remove the non-homologous tails from the 3′ ends of invading strands (Schiestl and Prakash, 1988, 1990; Adair et al., 2000; Sargent et al., 2000), reminiscent of the function of the endonuclease complex in SSA. It is more difficult to propose this function to explain the importance of the complex in gene targeting of the ends-out construct during TGR. This role of the XPF-ERCC1 complex for ends-out gene targeting in *P. patens* is of particular interest and should be consider in the light of other observations in other models. Indeed, in Arabidopsis ERCC1 has been proposed to be involved not only in SSA recombination as measured by a plasmid assay, but also in gene conversion/crossing over in chromosomal DNA (Dubest et al., 2004) and in mouse cells this complex is essential for ends-out gene targeting in the absence of non-homologous overhangs (Niedernhofer et al., 2001), implying a more general role for this endonuclease in recombination than the removal of non-homologous DNA overhangs from recombination intermediates. The endonuclease complex has also been shown to be important for TGR using ends-out targeting constructs in *S. cerevisiae* and separate studies have reported TGR efficiency decrease, ranging from a 3- to 40-fold reduction in *rad1* and *rad10* mutants (Schiestl and Prakash, 1988, 1990; Schiestl et al., 1994; Saparbaev et al., 1996; Symington et al., 2000; Langston and Symington, 2005). When using a classical ends-out gene targeting construct one possible role for the endonuclease could be the resolution of the large loop of mismatches that forms between the targeted gene and the heterologous selectable marker (that separate de 5′ and 3′ regions of homology of an ends-out construct) during the two ends invasion process of TGR (Niedernhofer et al., 2001; Langston and Symington, 2004, 2005). We could show here that the importance of the *P. patens* XPF-ERCC1 complex for targeted integration of the ends-out construct is not affected by the presence or the absence of an heterologous selectable marker.

In order to better understand the role of the *P. patens* XPF-ERCC1 complex in TGR using an ends-out construct we have compared the nature of the TGR events found in the wild type and in the mutants. As observed previously (Kamisugi et al., 2006), we could identify several types of gene targeting event (Supplementary Figure S6): (i) TGR, in which the targeted locus is replaced by a single copy of the transforming DNA (HR/HR) (ii) this may involve insertion of multiple copies (concatemers) of the targeting construct and (iii) “one-end gene targeting” or TGI, that may result from an homologous recombination event at one end of the construct accompanied by an apparent non-homologous end-joining event at the other (HR/NHEJ) (iv) this process may also involve insertion of multiple copies of the targeting construct. We could show here that the XPF-ERCC1 complex is more specifically involved in the mechanism that results in the integration of the targeting construct via TGI and in the integration of concatemers. The decrease in the number of concatemers and TGI events in the *xpf* or *ercc1* mutants context could explain, at least for a part, the general decrease in gene targeting efficiency observed using a ends-out type construct in absence of the XPF-ERCC1 complex. One hypothesis to explain the role of the complex in the formation of TGI and concatemers events could be its potential role in the handling of the looped-out heteroduplex intermediates (Supplementary Figure S6) formed during the invasion process in presence of the concatemers that are produced before integration at the targeted *APT* locus. In this context, and taking into consideration our recent data showing the involvement of the POLQ protein in TGI events formation in *P. patens* (Mara et al., 2019), it would be interesting to check for a potential interaction between the XPF-ERCC1 complex and the Alt-EJ pathway for the targeted integration of ends-out construct in this moss. Such a cross-talk between the RAD1-RAD10 complex and the Alt-EJ repair pathway, that, like the SSA pathway, involves the removal of non-homologous 3′ tails, has already been proposed in yeast and more recently in animals (Ma et al., 2003; Sallmyr and Tomkinson, 2018).

We have shown here an essential role of the XPF-ERCC1 endonuclease complex in genetic stability of the model plant *P. patens*. Moreover, we have shown for the first time in plants, the implication of this endonuclease in gene targeting through ends-in or ends-out constructs. If the role of this complex in ends-in construct integration can be easily explained by the capacity of this endonuclease to remove non-homologous 3′ ends tails the exact role of the complex in integration of ends-out type constructs is still puzzling and further work is needed. Different functions of the XPF-ERCC1 complex and at different steps of the process of targeted integration of ends-out constructs could be involved. These functions could be shared by other organisms, like yeast and animal cells, where this endonuclease has also been shown to be important. However, more specific roles, due to specificities of the mechanism of targeted integration in the different species, could be involved. One of these could consist in the removal of the “apparent” heterologous regions formed during concatemers production in *P. patens*. Existence of this putative mechanism in other species, and especially in flowering plants is an open question and one must take into consideration that *P. patens* has an intrinsic high level of homologous recombination that could lead to functions of the XPF-ERCC1 complex that would be specific to this moss. Nevertheless, deciphering of the shared and specific roles of the XPF-ERCC1 complex in integration of the ends-out type constructs in different species is important to better understand the action of this endonuclease in genome maintenance and could have also potential applications in order to improve the efficiency and/or the quality of gene targeting for applied research.

## Data Availability

All datasets generated for this study are included in the manuscript and/or the Supplementary Files.

## Author Contributions

FN, DS, AG-D, and J-MN designed the research. AG-D and PR performed the research with the help of FC, AE, and DS. FN, AG-D, DS, and J-MN wrote the manuscript with contributions from all the authors.

## Conflict of Interest Statement

The authors declare that the research was conducted in the absence of any commercial or financial relationships that could be construed as a potential conflict of interest.

## Footnotes

1 https://phytozome.jgi.doe.gov
