# Supplementary material for: The XPF-ERCC1 Complex Is Essential for Genome Stability and Is Involved in the Mechanism of Gene Targeting in Physcomitrella patens
Source: Front Plant Sci. 2019 May 9;10:588. doi: 10.3389/fpls.2019.00588 (PMC6521618; doi:10.3389/fpls.2019.00588)
Supplement: Supplementary file 1 [file Data_Sheet_1.pdf]

Table S1 Primers used in this study.

Locus	Primer name	orientation	Sequence 5’-3’
XPF	PpXPF#1	Forward	CTGGACGACAATTCCGATTA
	PpXPF#2	Reverse	TTTCTCCTGGTCATCTTCTG
	PpXPF#12	Reverse	AGCGCTGACCCTGTCAACCTT
	PpXPF#13	Forward	AGGTCCTTTCATGCTAGAGTG
ERCC1	PpERCC1#1	Forward	GTAGCACAGAAGGACAACAC
	PpERCC1#2	Reverse	GCTTGCCATGTCTGCTATTC
	PpERCC1#17	Forward	GATGTGATCAAACCGCTAC
	PpERCC1#18	Reverse	GCTTGCCATGTCTGCTATTC
APT	PpAPT#2	Forward	TTTTTGCGCTCGCTGTTTCTG
	PpAPT#14	Forward	AGATGTCGGCCTCCAAGGATG
	PpAPT#19	Reverse	CCCGACAACTTCTCACGACCC
	PpAPT#20	Reverse	TAAATAATTCTGACCCAAAGT
HygR	ProRev	Reverse	GTCTTGCGAAGGATAGTGGG
	TerFwd	Forward	CGCTGAAATCACCAGTCTCTCT

Table S2 Comparison of transformation and gene targeting efficiencies using an ends-out type targeting construct containing no heterologous selectable marker.

Type of transforming DNA	Genotypes	Regenerants [a]	$2FA^R$ Plants [b]	Gene targeting frequency (%) [c]
Ends-out	Wild type	359848	2584	0.72 ± 0.13 [d]
Homologous ends	*xpf*	464812	2026*	0.43 ± 0.08
	ercc1	200385	1042*	0.52 ± 0.07
Ends-out	Wild type	54667	82	0.15 ± 0.03
Heterologous ends	*xpf*	62500	75	0.12 ± 0.04
	ercc1	56364	62	0.11 ± 0.07
Ends-in	Wild type	30682	183	0.59 ± 0.03
Homologous ends	*xpf*	36667	176**	0.48 ± 0.03
	ercc1	49524	208*	0.42 ± 0.04
Ends-in	Wild type	72857	102	0.14 ± 0.01 [d]
Heterologous ends	*xpf*	54286	38*	0.07 ± 0.02
	ercc1	87013	87**	0.1 ± 0.02

[a] Protoplasts were transformed with the different vectors (see Fig. 3 and Fig.4) and regenerants were selected on 2-fluoroadenine.
[b] $2\text{-}FA^R$ clones are the clones that experienced a gene targeting event and thus survived after subculture on 2-FA medium. Differences between wild type and the mutants were compared using Fisher's exact test. * Correspond to p value <0.01, ** Correspond to p value <0.05.
[c] GT frequency (in %) express the frequency of 2-FA resistant among the population of regenerants.
[d] Average and standard deviation were determined from at least 3 independent experiments.
