# Supplementary material for: The XPF-ERCC1 Complex Is Essential for Genome Stability and Is Involved in the Mechanism of Gene Targeting in Physcomitrella patens
Source: Front Plant Sci. 2019 May 9;10:588. doi: 10.3389/fpls.2019.00588 (PMC6521618; doi:10.3389/fpls.2019.00588)
Supplement: Supplementary file 2 [file Data_Sheet_2.pdf]

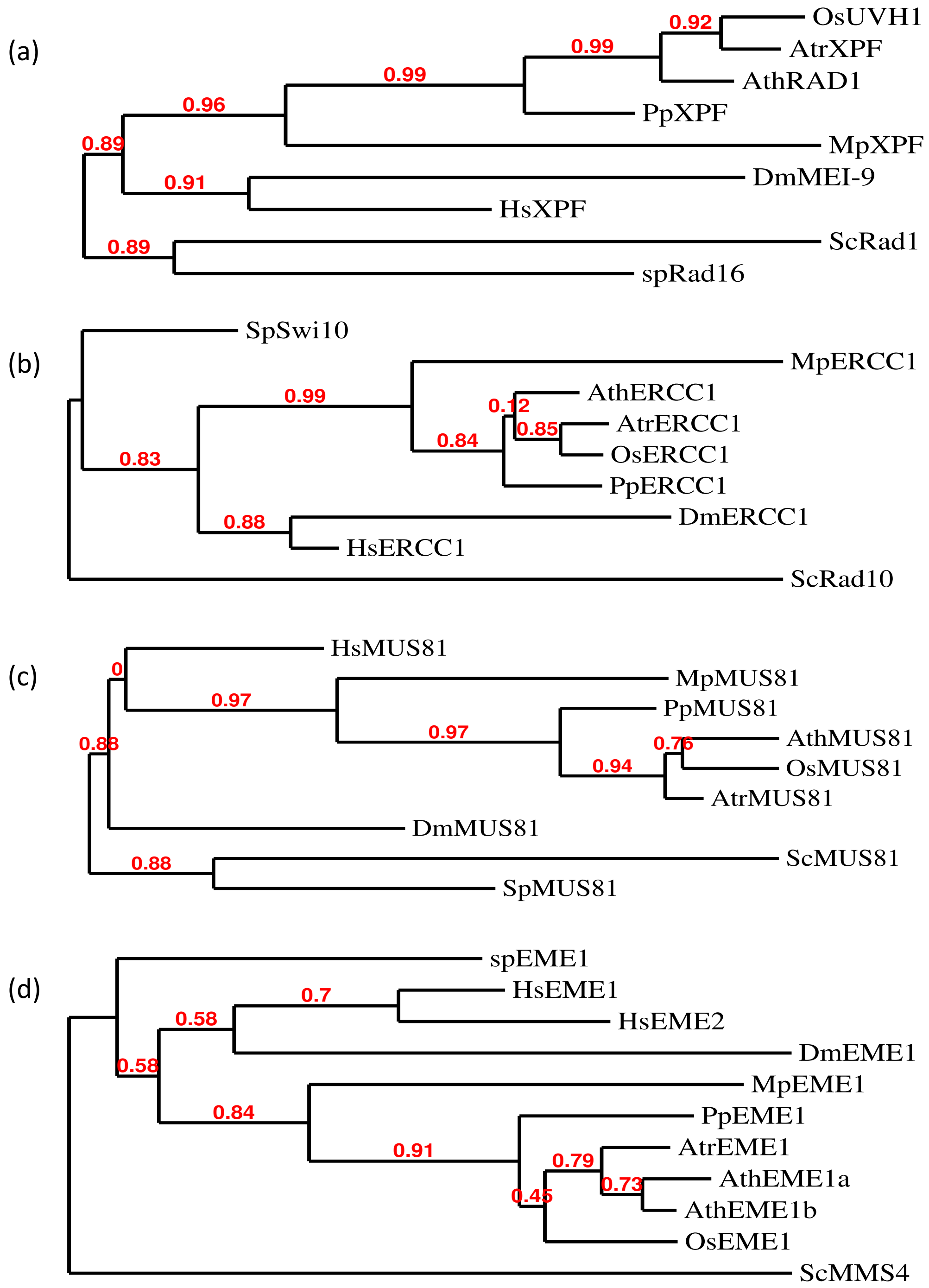

Figure S1. Physcomitrella genome encodes homologs of ERCC1 and XPF. The dendrograms illustrate the sequence relationships between several XPF (a), ERCC1 (b), MUS81 (c) and EME1 (d) proteins within the XPF super family of structure specific endonucleases. The branch lengths are proportional to the sequence divergence.

Accession numbers used in this analysis are, for *Physcomitrella patens*: PpXPF (XP_024403241), PpERCC1 (XP_024378954), PpMUS81 (XP_024398365), PpEME1 (XP_024376616), for *Arabidopsis thaliana*: AthXPF (NP_001031991), AthERCC1 (NP_187172), AthMUS81 (NP_194816), AthEME1a (NP_001189572), AthEME1b (NP_179804), for *Oryza sativa*: OsUVH1 (XP_015630768), OsERCC1 (XP_015612964), OsMUS81 (XP_015622535), OsEME1 (XP_015637261), for *Amborella trichopoda*: AtrXPF (XP_020519892), AtrERCC1 (XP_020525873), AtrMUS81 (XP_020522223), AtrEME1 (XP_020530544), for *Micromonas pusilla*: MpXPF (XP_003058408), MpERCC1 (XP_003056392), MpMUS81 (XP_003055357), MpEME1 (XP_003060706), for *Drosophila melanogaster*: DmMEI9 (AAC46917), DmERCC1 (NP_477468), DmMUS81 (NP_569873), DmEME1(), for *Homo sapiens*: HsXPF (NP_005227), HsERCC1 (NP_001974), HsMUS81 (AAL28065), HsEME1 (NP_689676), HsEME2 (NP_001244299), for *Saccharomyces cerevisiae*: ScRad1 (AJW05769), ScRad10 (NP_013614), ScMus81 (EDN60717), ScMms4 (CBK39173), for *Schizosaccharomyces pombe*: SpRad16 (NP_587855), ScSwi10 (NP_596115), SpMus81 (NP_001343019), SpEme1 (NP_594132).

```
SpombeRad16     VNTRIAGGGQLSITNEKPRVIVDLREFRSSLPSILHGNNFSVIPCQLLVGDYILSPKICV  693
HomoXPF         TDTRKAGG--QEQNGTQQSIVVDMREFRSELPSLIHRRGIDIEPVTLEVGDYILTPEMCV  724
PhyscoXPF       LDTRKGVG--RKQAQKQMQVVVDMREFGSSLPSVLHQQGMKILPVTLEVGDYILSPDICV  857
AthalXPF        SLTRKAGG--RKELEKETQVIVDMREFMSSLPNVLHQKGMKIIPVTLEVGDYILSPSICV  766
                  ** . *   .    :  ::**:*** *.**.::* . :.: *  * ******:*.:**

SpombeRad16     ERKSIRDLIQSLSNGRLYSQCEAMTEYYEIPVLLIEFEQHQSFTSPPFSDLSSEIGKNDV  753
HomoXPF         ERKSISDLIGSLNNGRLYSQCISMSRYYKRPVLLIEFDPSKPFSLTSRGALFQEISSNDI  784
PhyscoXPF       ERKSIADLFSSFSSGRLYHQAETMSRYYKYPVLLIEFSQDKSFSLQAASDIGEDIAPANI  917
AthalXPF        ERKSIQDLFQSFTSGRLFHQVEMMSRYYRIPVLLIEFSQDKSFSFQSSSDISDDVTPYNI  826
                ***** **: *:..***: *   *:.**. *******.  : *:    . : .::   ::

SpombeRad16     QSKLVLLTLSFPNLRIVWSSSAYVTSIIFQDLKAMEQEPDPASAASIGLEA------GQD  807
HomoXPF         SSKLTLLTLHFPRLRILWCPSPHATAELFEELKQSKPQPDAATALAITADSE-----TLP  839
PhyscoXPF       ISKLSLLVLHFPRLRIVWSRSLHATADIFMALKSNQNEPDLDRAMRVGVPTEDGLIEGDI  977
AthalXPF        ISKLSLLVLHFPRLRLLWSRSLHATAEIFTTLKSNQDEPDETRAIRVGVPSEEGIIENDI  886
                 *** **.* **.**::*. * :.*: :*  **  : :**   *  :   :

SpombeRad16     STNTYNQAPLDLLMGLPYITMKNYRNVFYGGVKDIQEASETSERKWSELIGPEA-GRRLY  866
HomoXPF         ESEKYNPGPQDFLLKMPGVNAKNCRSLMH-HVKNIAELAALSQDELTSILGNAANAKQLY  898
PhyscoXPF       RAENFNTTAVELLRRLPGVSDANYRSLMA-GCKSIAEMALLSVDELAELMGGKQPARMLR  1036
AthalXPF        RAENYNTSAVEFLRRLPGVSDANYRSIME-KCKSLAELASLPVETLAELMGGHKVAKSLR  945
                 ::.:*    ::*  :* :.  * *.::    *.: * :       :.::*    .: *

SpombeRad16     SFFRKQLKDYE-------    877
HomoXPF         DFIHTSFAEVVSKGKGKK    916
PhyscoXPF       EFLDAKCPTLV-------    1047
AthalXPF        EFLDAKYPTLL-------    956
```

Figure S2. Physcomitrella genome encodes an XPF homolog. Clustal Omega alignment of the 250 C terminal amino acids of XPF proteins from *P. patens*, *A. thaliana*, *S. pombe* and *H. sapiens*. The restriction endonuclease type II-like domain or ERCC4 domain (pfam02732 (819-946) and the RuvA 2-like domain (978-1042) or HhH domain (SMART **HHH_5** 987-1040) are indicated with green and blue lines, respectively. Green box contains the residues required for nuclease activity. Accession numbers used in this analysis are as follows: PpXPF (P_024403241), AtXPF (NP_001031991), SpRAD16 (NP_587855) and HsXPF (NP_005227).

```
SpomSWI10       ------------------------------------------------------------   0
HomoERCC1       MD----PGKDKE----------GVPQPSGPPARKKFVIPLDEDEVPPGVAKPLFRSTQSL   46
PpatERCC1       MAAREVDSQAGN----------GMKSFLHIPSHAEVLERSRP-QAPPSFFRPSSSAAPPV   49
AthalERCC1      MANEDDDGEKSRSLHQQIARKPKTQIVIGVPSYQEVLESSQTKSTPPSLFKP--------   52

SpomSWI10       --------------------------------MSDIDDEEF-------------------   9
HomoERCC1       ---------------------------------PTVDTSA----------Q---AAPQT-   59
PpatERCC1       EAVGAASVSSSASQQQQSLGRAGGVPGSFGEAFSFVRQTEFYTPPPAAPTPTVPVAPSEG   109
AthalERCC1      -------------------------SQSFSQAFAFVKSSDVYSPPPPSSAAASSSQPSGA   87
                                                   :

SpomSWI10       -----------EQLAVSALEEVEKKAGFAQQPTPQKVSRVTAHSILVNPRQKGNPLLPHV   58
HomoERCC1       -YAEYAISQPLEGAGATCPTGSEPLA--GETPNQALKPGAKSNSIIVSPRQRGNPVLKFV   116
PpatERCC1       ----------VPGNGVG------SRS----QEALPTVNSQARNAILVNRRQTGNPVLKYI   149
AthalERCC1      SQVPHSSSQTHQTDGASSS--STPVA----TGSVPSNTTQNRNAILVSHRQKGNPLLKHI   141
                              ..         :                .:*:*. ** ***:* .:

SpomSWI10       RNVPWEYTDIVPDFVMGTGICSLFLSLKYHHLHPEYIYSRISKLGKSYNLRILLILVDVE   118
HomoERCC1       RNVPWEFGDVIPDYVLGQSTCALFLSLRYHNLHPDYIHGRLQSLGKNFALRVLLVQVDVK   176
PpatERCC1       RNVRWVFGDIVPDYLLGQSTCALYISLRYHLLHPDYLYFRIRELQKGFRLRVVLCHVDLE   209
AthalERCC1      RNVKWVFSDIIPDYVLGQNSCALYLSLRYHLLHPDYLYFRIRELQKNFKLSVVLCHVDVE   201
                *** * : *::**:::*   *:*::**:** ***:*:: *: .* * : * ::*  **::

SpomSWI10       NHQASIQELVKTSIVNQYTLILAWSSEEAARYLETYKAYENMSPALIMEKPSTDYLSQVQ   178
HomoERCC1       DPQQALKELAKMCILADCTLILAWSPEEAGRYLETYKAYEQKPADLLMEKLEQDFVSRVT   236
PpatERCC1       DVIKPLHEVIKTSLLHDCSLLCAWSLEECARYLETLKTYEHKPADNIQERTDNDYISRMT   269
AthalERCC1      DTVKPLLEVTKTALLHDCTLLCAWSMTECARYLETIKVYENKPADLIQGQMDTDYLSRLN   261
                :    : *: * .:: : :*: ***  *..***** *.**.     :  : . *::*::

SpomSWI10       SFLTSIRGINKSDSLSLLSKFGSLERALVASRDELEQLEGWGPTKVNRFLEAVQQPFMSH   238
HomoERCC1       EQLTTVKSVNKTDSQTLLTTFGSLEQLIAASREDLALCPGLGPQKARRLFDVLHEPFLKV   296
PpatERCC1       SALTTVRHVNKTDVLTLGSTFETLTGIFSASMEDLARCPGIGERKVKRLYDAFHEPFRRT   329
AthalERCC1      HSLTSIRHVNKSDVVTLGSTFGSLAHIIDASMEDLARCPGIGERKVKRLYDTFHEPFKRA   321
                  **::: :**:*  :* :.* :*   : ** ::*    * *  *..*: :..::**

SpomSWI10       STIKRPEAINLKQT----------------------------------------------   252
HomoERCC1       P-----------------------------------------------------------   297
PpatERCC1       TKQLQLDNMLTKVSQSVTAES--NTTLDSSTMQIDTVDTAVGASEPNADIPELSVRQALN   387
AthalERCC1      TSSYPSVVEPPIPEAPVEKDVNSEEPVEED---EDFVED-SRKRKKKEPEPEKTVKTALS   377

SpomSWI10       ----------------------------------------   252
HomoERCC1       ----------------------------------------   297
PpatERCC1       TAYAKVVERRRGSRTETSMKDMSTDCRHTPLVNVEVIDIT   427
AthalERCC1      AVFARYSDRLSKKKEKQKEKDTTTASDAETHQN-------   410
```

Figure S3. Physcomitrella genome encodes an ERCC1 homolog. Clustal Omega alignment of ERCC1 sequences from moss (Ppat), Arabidopsis (Athal), Human (homo) and fission yeast (Spom). Restriction endonuclease type II-like domain (132-257), RuvA 2-like domain (259-328) and Helix-hairpin-helix DNA-binding motif (305-321) are indicated with green, blue and red lines respectively. Hydrophobic amino acids critical for the formation of the HhH motifs are indicated with brown boxes. Accession numbers used in this analysis are as follows: PpERCC1 (XP_024378954), AtERCC1 (NP_187172), Spombe SWI10 (NP_596115) and HsERCC1(NP_005227).

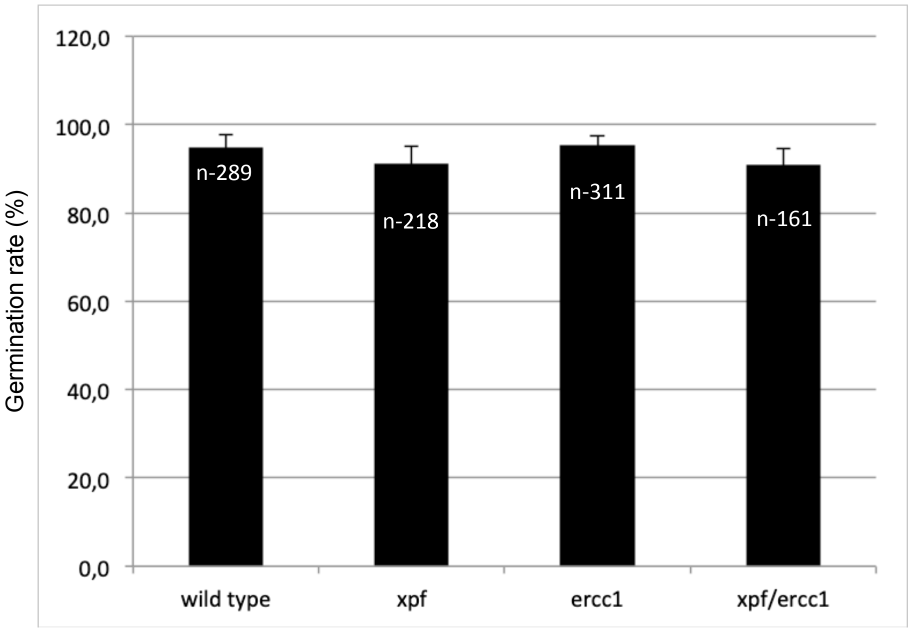

Figure S4. Germination rate of wild type *and xpf*, *ercc1* and *xpf/ercc1* mutants spores. Freshly harvested spores from wild type *and xpf*, *ercc1* and *xpf/ercc1* capsules were sown on PpNH4 medium. Percentages of germination were calculated three days after sowing. Error bars indicate SD based on three independent experiments.

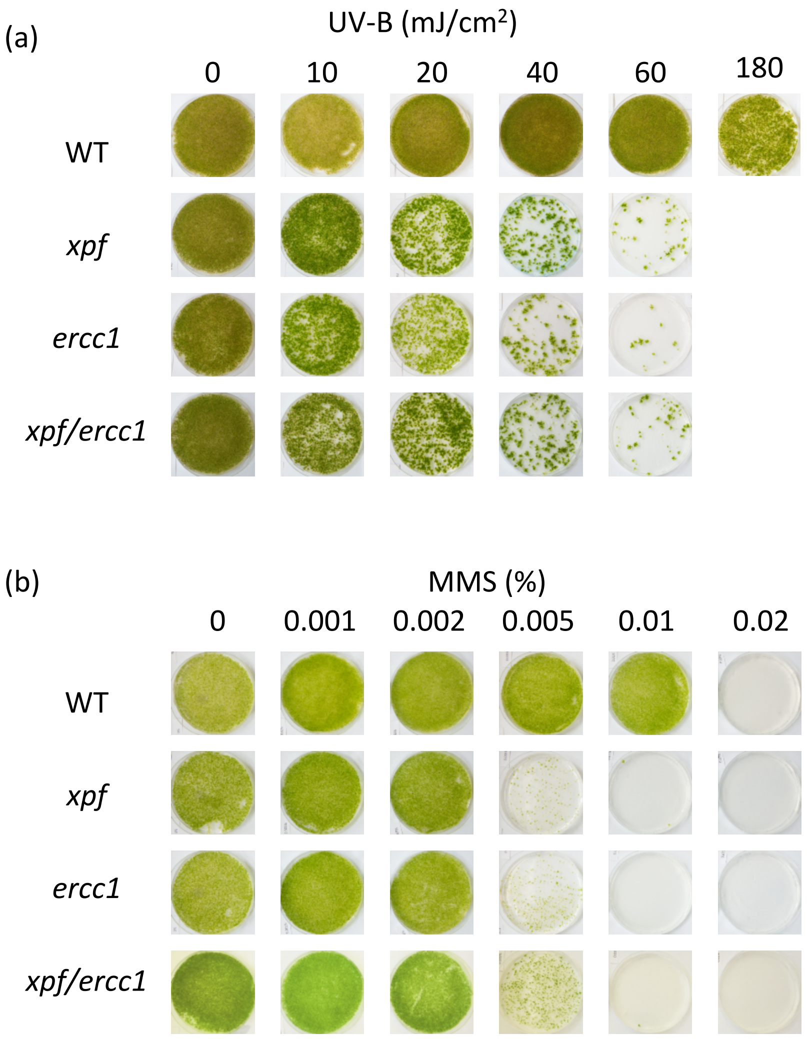

Figure S5. Sensitivity of the wild-type (WT) and of the *xpf*, *ercc1* and *xpf/ercc1* mutants towards genotoxic agents. 3 weeks old plates of WT and the 3 mutants exposed to low doses of UV-B light B (a) or to increasing doses of MMS (b).

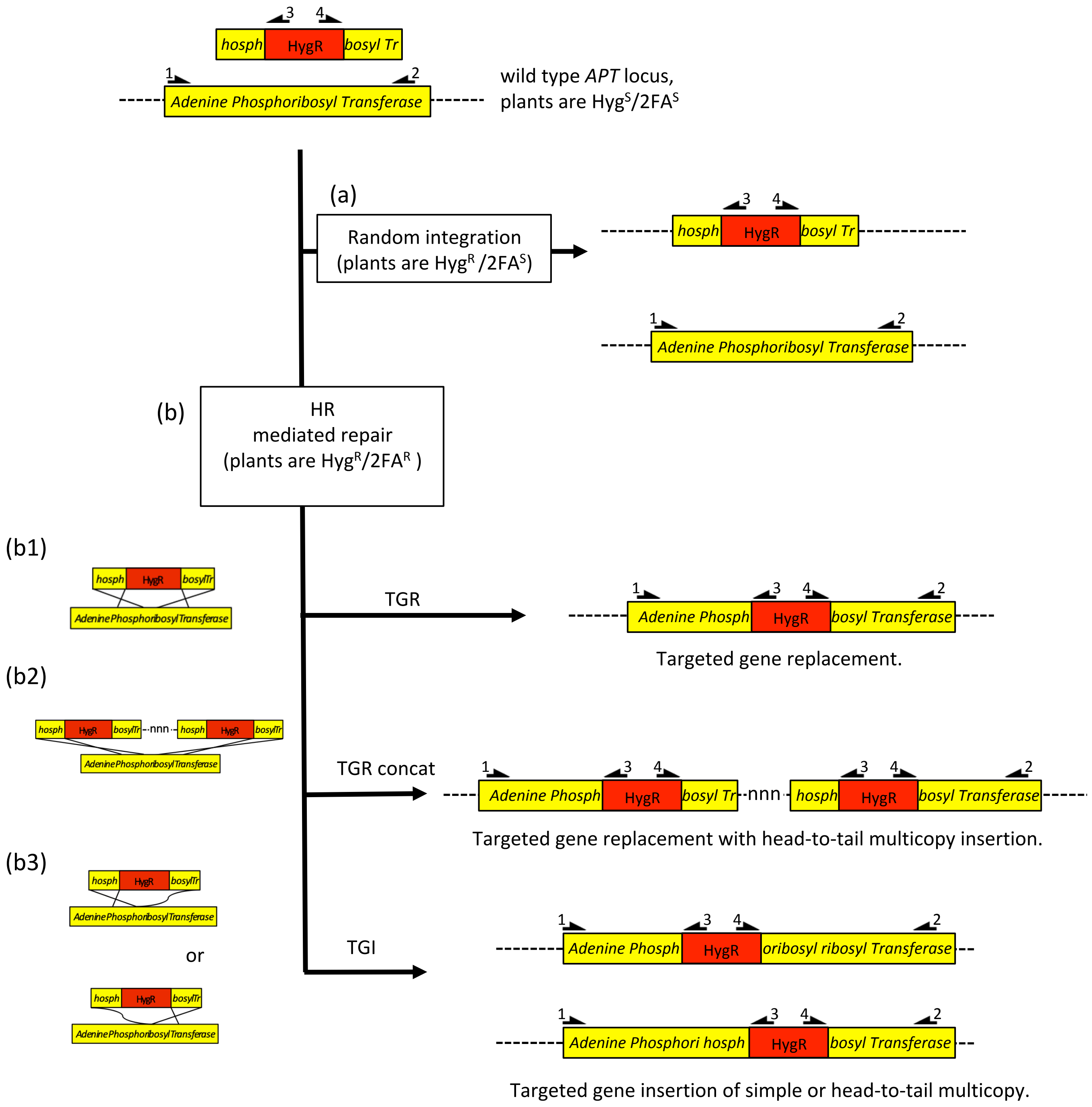

Figure S6. Different outputs of repair of a CRISPR-Cas9 induced DSB in presence of a donor template. Integration of the Donor DNA can be random (a) and in this case plants are resistant to hygromycine but sensitive to 2FA, or via gene targeting (b). As targeting of the *APT* gene leads to resistance to 2FA, selection of hygromycin resistant plants on this compound will permit selection of clones that experienced an HR mediated integration of the donor DNA at the targeted locus. HR mediated integration of the donor template can lead to TGR (b1), TGR with concatemers (b2) or TGI (b3). For a given 2FA and Hygromycin resistant clone, analysis of PCR products 1+3 and 4+2 will permit to determine whether it results from a TGR or TGI integration. Furthermore analysis of PCR products 1+2 will permit to determine whether it is or it is not a monocopy insertions. In this study primers 1, 2, 3 and 4 correspond to PpAPT#2, PpAPT#20, ProRev and TerFwd respectively (Table S1).
